# Intraoperative Transfusion is Independently Associated with a Worse Prognosis in Resected Pancreatic Cancer—A Retrospective Cohort Analysis

**DOI:** 10.3390/jcm9030689

**Published:** 2020-03-04

**Authors:** Si Youn Kim, Munseok Choi, Ho Kyoung Hwang, Seoung Yoon Rho, Woo Jung Lee, Chang Moo Kang

**Affiliations:** 1 Yonsei University College of Medicine, Seoul 03722, Korea; siyoun88@naver.com; 2 Division of Hepatobiliary and Pancreatic Surgery, Department of Surgery, Yonsei University College of Medicine, Seoul 03722, Korea; CMS2598@yuhs.ac (M.C.); DRHHK@YUHS.AC (H.K.H.); FORSH7@yuhs.ac (S.Y.R.); wjlee@yuhs.ac (W.J.L.); 3 Pancreatobiliary Cancer Center, Yonsei Cancer Center, Severance Hospital, Seoul 03722, Korea

**Keywords:** transfusion, pancreatic cancer, blood loss, survival rates, intraoperative

## Abstract

Backgrounds: Investigate whether intraoperative transfusion is a negative prognostic factor for oncologic outcomes of resected pancreatic cancer. Methods: From June 2004 to January 2014, the medical records of 305 patients were retrospectively reviewed, who underwent pancreatoduodenectomy, pylorus preserving pancreatoduodenectomy, total pancreatectomy, distal pancreatectomy for pancreatic cancer. Patients diagnosed with metastatic disease (*n* = 3) and locally advanced diseases (*n* = 15) were excluded during the analysis, and total of 287 patients were analyzed. Results: The recurrence and disease-specific survival rates of the patients who received intraoperative transfusion showed poorer survival outcomes compared to those who did not (*P* = 0.031, *P* = 0.010). Through multivariate analysis, T status (HR (hazard ratio) = 2.04, [95% CI (confidence interval): 1.13–3.68], *P* = 0.018), N status (HR = 1.46 [95% CI: 1.00–2.12], *P* = 0.045), adjuvant chemotherapy (HR = 0.51, [95% CI: 0.35–0.75], *P* = 0.001), intraoperative transfusion (HR = 1.94 [95% CI: 1.23–3.07], *P* = 0.004) were independent prognostic factors of disease-specific survival after surgery. As well, adjuvant chemotherapy (HR = 0.67, [95% CI: 0.46–0.97], *P* = 0.035) was independently associated with tumor recurrence. Estimated blood loss was one of the most powerful factors associated with intraoperative transfusion (*P* < 0.001). Conclusions: Intraoperative transfusion can be considered as an independent prognostic factor of resected pancreatic cancer. As well, it can be avoided by following strict transfusion policy and using advanced surgical techniques to minimize bleeding during surgery.

## 1. Background

Pancreatic cancer is an aggressive disease with poor prognosis. Due to its tendency to remain asymptomatic until it reaches an advanced stage, it is difficult to diagnose and typically deemed unresectable at the time of diagnosis. When deemed operable, pancreatectomy is considered as the best option in terms of longer survival [1]. Pancreatectomy in this article refers to all types of curative-intent surgery for pancreatic cancer which includes pancreatoduodenectomy, pylorus preserving pancreatoduodenectomy, total pancreatectomy and distal pancreatectomy. However, even when some pancreatic cancers are discovered at a potentially operable stage, pancreatectomy survival rate is very poor compared to other types of resected cancers [2].

Factors concerning poor prognosis of pancreatic cancers include high level of CA19-9, large tumor size, lymph node metastasis, and perineural and lymphovascular invasion [3,4,5,6]. However, these factors cannot be controlled by surgeons and occur before pancreatectomy is performed. Thus, instead of examining such uncontrollable prognostic factors, we aim to identify the factors that can be controlled either at the time of or after surgery. Although adjuvant chemotherapy and radiotherapy can be performed after surgery, we decided to manipulate the timeline of surgery. Factors such as availability of combined resection, operation time, amount of blood loss, and availability of transfusion are decided at the time of surgery and could play a role in improving survival and recurrence rates of patients who undergo pancreatectomy. Of these factors, we focused on transfusion and their inappropriate performance during pancreatectomy and hypothesized that reduction of such inappropriate transfusions could improve patient outcomes [7]. Given that the interactions of the host immune system and cancer microenvironment play an important role in determining outcome, and the fact that transfusion lowers a patient’s immunity and aggravates outcome [8,9], we concluded that intraoperative transfusion (IOT) might lead to poor outcome.

There have been many studies underlining the poor effects of transfusion performed during pancreatic cancer surgery on patient outcomes. Most, however, focus on the effect of IOT on recurrence and survival in pancreaticoduodenectomy cases and not in other types of pancreatectomy, because of the complicated procedure of pancreaticoduodenectomy [10,11,12]. One particular study suggests that transfusion performed during pancreatectomy for left-sided pancreatic cancer patients is harmful [7]. While that study focused on more than just pancreaticoduodenectomy, it involved a small number of subjects and focused only on left-sided pancreatic cancer; therefore, it is unlikely to provide a strong guideline against transfusion during pancreatic cancer surgery. Thus, we performed our study on a larger number of subjects and widened the scope to involve all types of pancreatectomy. By doing so, we hope to provide strong evidence that IOT has a negative effect on the prognosis of patients undergoing pancreatectomy. The purpose of this study is to investigate the oncologic impact of IOT in all types of pancreatectomy.

## 2. Materials and Methods

This study was conducted in accordance with the Declaration of Helsinki and was approved by the Institutional Review Board of Severance Hospital (registered on 3 March 2019, and registration number is 2019-0526-001). All patients who underwent pancreatectomy in Severance Hospital of Yonsei University College of Medicine from June 2004 to January 2014 were enrolled in this study to evaluate the oncologic impact of IOT in all types of pancreatectomy. Patients diagnosed with metastatic disease (*n* = 3) and locally advanced diseases (*n* = 15) were excluded during the analysis, and total of 287 patients were analyzed. All patients’ medical records were retrospectively reviewed. We analyzed clinicopathological features, intra-operative findings (including combined resection of other organs or vascular structures), operation types, neo-adjuvant chemoradiation therapy, TNM (tumor, nodes and metastases) stage by 8th AJCC classification, postoperative complications, and survival outcomes. The patients with a pathologic diagnosis of only ductal adenocarcinoma were enrolled, and those with other pathologic conditions of the pancreas were excluded to create a homogenous patient population. IOT was defined as any amount of red blood cell transfusion received during the operative procedure. Details pertaining to the amount of bleeding during operation were evaluated based on operative medical records. Continuous variables are expressed as mean ± SD (standard deviation), and nominal variables are expressed as frequency (%). Comparative analysis was performed using the Chi-square test and Student’s *t*-test. Survival analysis was calculated using the Kaplan-Meier method, and the significance of a difference between groups was assessed with a log rank test. Multivariate analysis was performed to identify risk factors of cancer recurrence and survival rate using a Cox proportional hazards model. P-values less than 0.05 were considered statistically significant.

## 3. Results

### 3.1. General Characteristics of the Patients

During the study period, 287 patients underwent pancreatectomy for pancreatic ductal adenocarcinoma (Table 1). The 113 female patients (39.4%) and 174 male patients (60.6%) had an average age of 62.5 ± 9.5 years. Fifty-seven patients (19.9%) were asymptomatic. Eighty-one patients (28.2%) underwent neo-adjuvant chemotherapy prior to surgery, while another 206 patients (71.8%) underwent upfront surgery. Seventeen patients underwent PD (pancreatoduodenectomy) (5.9%), 162 patients underwent PPPD (Pylorus preserving pancreatoduodenectomy) (56.4%), 101 patients underwent DP (distal pancreatectomy) (35.2%), and 7 patients underwent TP (Total pancreatectomy) (2.4%). Combined resection was performed in 93 patients (32.4%). Two hundred and forty-nine patients (86.8%) underwent R0 resection. Post-operative adjuvant chemotherapy was provided in 211 patients (73.5%). Post-operative mortality occurred in 3 patients (1.0%), and patients with other complications were assorted according to Clavien-Dindo Classification. It was noted that 69 patients (24.0%) had IOT (Table 1).

### 3.2. Chronological Trend and Potential Adverse Oncologic Impact of IOT

**Figure 1:**
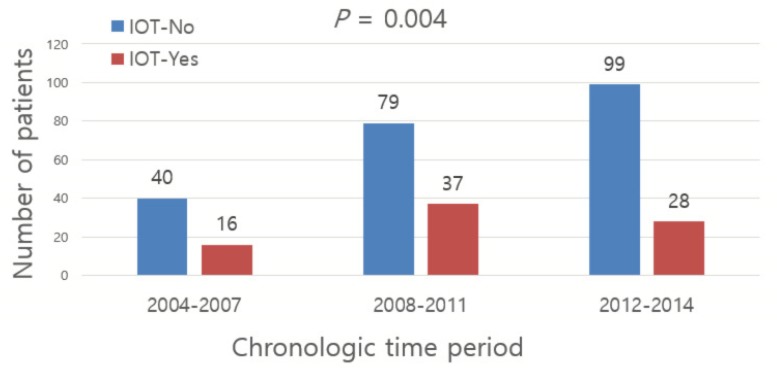
Trend of IOT (intraoperative transfusion) according to time period.

The incidence of IOT declined significantly during the time period (*P* = 0.004, Chi-square with linear-to-linear association, Figure 1). In the early period (2004~2007), 28.6% of the patients (16 out of 56 patients) who underwent radical pancreatectomy for pancreatic cancer received IOT; during the last period (2012–2014), IOT was performed in only 13.9% of the patients (16 out of 115 patients).

**Figure 2:**
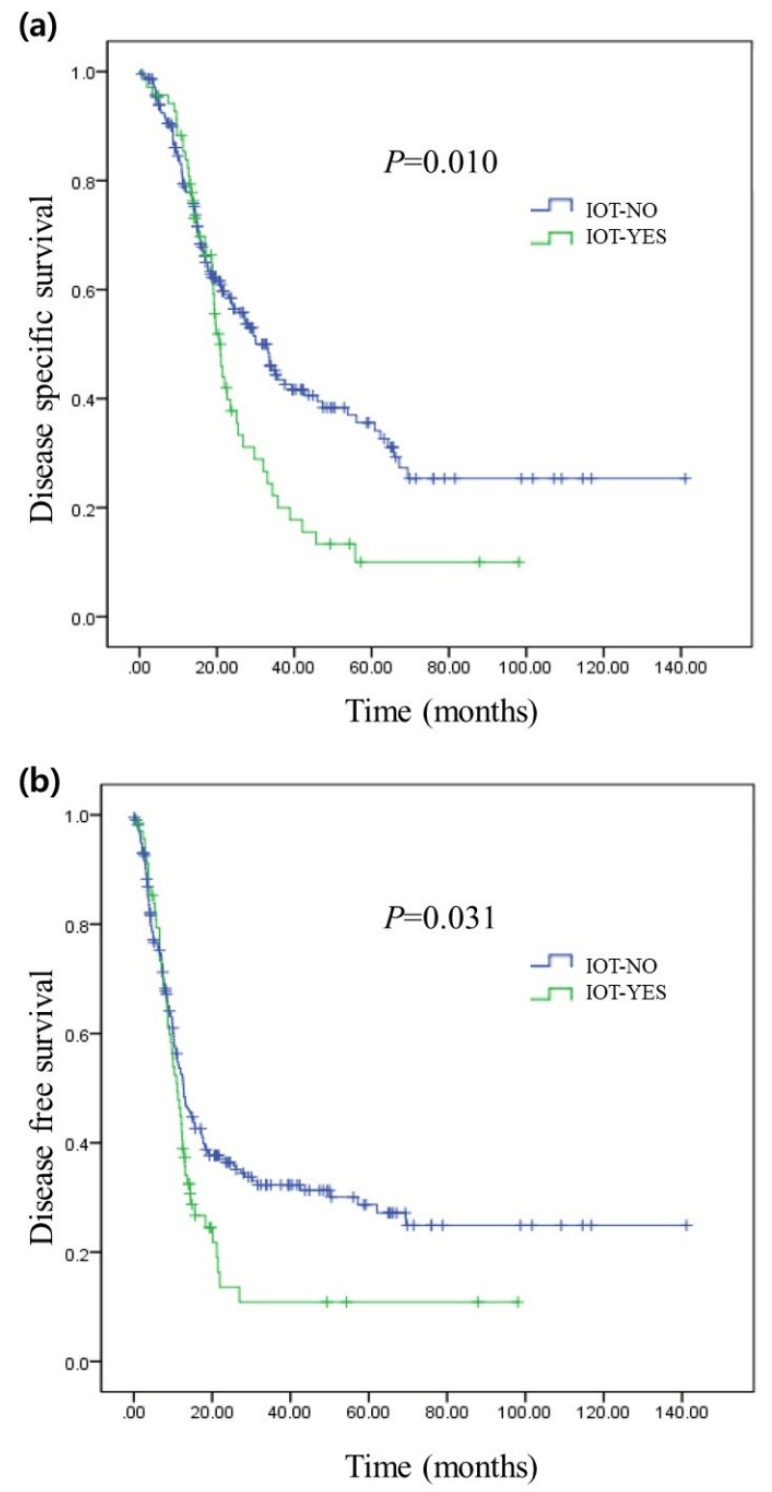
Long-term oncologic outcomes of resected pancreatic cancer according to IOT (**a**) represents the comparison of disease-specific survival rates between the patients who received IOT and those who did not. (**b**) represents the comparison of recurrence rates between the patients who received IOT and those who did not.

The disease-specific survival rate of the patients who received IOT showed was significantly poorer compared to that of the group of patients that did not undergo transfusion during surgery (median survival of 20 months (95% CI: 18–22) vs. median survival of 33 months (95% CI: 27–38), *P* = 0.010, Figure 2a). The recurrence rate also showed a significant difference between the two groups, with *P* = 0.031. The median recurrence interval of patients who underwent IOT was 11 months (95% CI: 8–13), and that of patients who did not undergo IOT was 12 months (95% CI: 10–15, Figure 2b).

### 3.3. Determining Prognostic Factors in Resected Pancreatic Cancer

In univariate analysis (Table 2), IOT (HR = 1.41 [95% CI: 1.03–1.94], *P* = 0.031) was associated with tumor recurrence. Also, IOT (HR = 1.55 [95% CI: 1.10–2.17], *P* = 0.011), T status (T3&T4, HR = 1.75 [95% CI: 1.04–2.95], *P* = 0.033), N status (N1&N2, HR = 1.65 [95% CI: 1.20–2.26], *P* = 0.002) were associated with disease-specific survival rate in resected pancreatic cancer.

Multivariate analysis was performed on factors with p-value less than 0.1 in univariate analysis and was adjusted with the following cofounding factors; IOT, gender, NeoCRT, Adj. CTx, combined resection, complications, T status, N status, R status, past history, operation time, age, symptoms, preoperative CA19-9 level, EBL, tumor location, resectability, combined resection, complications, surgery type, LVI, PNI, and the year surgery was performed. The analysis showed that T status (HR = 2.04, [95% CI: 1.13–3.68], *P* = 0.018), N status (HR = 1.46 [95% CI: 1.00–2.12], *P* = 0.045), Adj.CTx (HR = 0.51, [95% CI: 0.35–0.75], *P* = 0.001), IOT (HR = 1.94 [95% CI: 1.23–3.07], *P* = 0.004), were independent prognostic factors of disease-specific survival after surgery (Table 3). As well, Adj.CTx (HR = 0.67, [95% CI: 0.46–0.97], *P* = 0.035) was independently associated with tumor recurrence (Table 3).

### 3.4. Predicting IOT in Resected Pancreatic Cancer

It was analyzed that combined organ resection (*P* < 0.001), and EBL (*P* < 0.001) and the year surgery was performed (*P* = 0.004) were significantly related to IOT. Therefore, IOT group correlated with higher amount of EBL. However, there were no significant differences between age, gender, past history, symptoms, complications between the group that went through IOT and the group that did not. (*P* > 0.05) Additionally, differences between the two groups regarding T status, N status, M stage, neoadjuvant chemoradiation therapy, adjuvant chemotherapy, tumor location, lymphovascular invasion, perineural invasion, resectability of tumors, surgery types, operation time, curative resection between the two groups were also analyzed: there was no differences the two groups. (*P* > 0.05).

## 4. Discussion

There have been several studies [13,14,15] concerning methods to improve surgical outcomes of resected pancreatic cancer during the past years, though none have achieved great success. The 5-year survival rate of overall pancreatic cancer is still 9%, and even for the small portion of people who are diagnosed with localized tumor and deemed operable, the 5-year survival is only 37% which is very poor compared with other types of tumors [2,16].

Although factors that have an influence on resected pancreatic patient survival and tumor recurrence rates have been defined by many researchers [3,4,5,6,17], there are few studies concerning factors changeable by surgeons during the intraoperative timeline. Our study showed that T status, N status, and Adj.CTx were independent prognostic factors associated with tumor recurrence and disease specific survival rates in resected pancreatic cancer (Table 3). However, the importance of this study is that IOT can be considered as an independent prognostic factor associated with survival outcomes of resected pancreatic cancer (*P* = 0.004, Table 3).

Many studies have revealed the negative effect of blood transfusion on patient survival in various kinds of resected cancers by showing patients receiving perioperative blood transfusions have a significantly worse prognosis than patients undergoing cancer surgery without a perioperative transfusion [18,19,20,21,22]. However, few studies have analyzed the impact of IOT on oncologic outcomes of pancreatectomy. A previous study reported by Hwang et al. [7] suggested that IOT in left-sided pancreatic cancer is a prognostic factor associated with tumor recurrence among patients who underwent pancreatectomy. However, their study focused only on left-sided pancreatic cancer and involved a relatively small number of subjects. Therefore, it remained controversial whether transfusion has a detrimental effect on resected pancreatic cancer.

Although it is hard to argue the idea that IOT is only the aftermath of the large blood loss which represents tumor’s size and surgery type, combined resection, and patient’s overall health and not the prognostic factor itself, by using multivariate analysis which was adjusted with many confounding factors, we were able to prove IOT is an independent prognostic factor of resected pancreatic cancer. As well, among the factors defined to have an impact on disease-specific survival of resected pancreatic cancer, IOT was the only factor that can be controlled by surgeons during the intraoperative period. Since EBL was significantly associated with transfusion during surgery (*P* < 0.001, Table 4), we concluded that surgeons should take precautions to reduce bleeding during pancreatectomy and thereby reduce the chance of IOT. To improve the oncologic outcomes of patients who undergo pancreatectomy, bleeding should be avoided by applying advanced surgical techniques based on anatomical knowledge, and inappropriate transfusion should be avoided by following a strict transfusion policy in consensus with the anesthesiology department. Also, although invasive pancreatectomy for pancreatic cancer is still controversial, based on the present observation that EBL was found to be the one of the significant factors contributing to IOT, minimally invasive pancreatectomy may have potential rationale in managing resectable pancreatic cancer. Many literature reviews and meta-analysis suggested that laparoscopic and robotic pancreatectomy were strongly associated with less EBL and less incidence of IOT [23,24,25,26,27,28,29,30].

However, our present study has several limitations. Firstly, our study was a single-center, retrospective study with a limited number of patients. If the study was conducted with more subjects in multi-center, the results would have been more powerful. Hence, further research is needed. By using broad clinical data from Korean multicenter research and Korea-Japan joint research, more powerful evidence for prognosis of transfusion in resected pancreatic cancer could be made. Also, through prospective cohort study of strict transfusion policy, a comparative study using propensity score matching can be envisioned. Secondly, our study failed to support an association between tumor recurrence and IOT. Although a Kaplan-Meier curve (*P* = 0.031, Figure 2b) and univariate analysis showed that IOT was strongly associated with tumor recurrence (*P* = 0.031, Table 2), subsequent multivariate analysis indicated that IOT is not accurately associated with tumor recurrence in resected pancreatic cancer (*P* = 0.056, Table 3). Again, with study conducted with more subjects in multi-center, the result may indicate IOT as a prognostic factor for tumor recurrence.

In summary, IOT was strongly associated with poorer survival outcomes of resected pancreatic cancer. Although the trend of performing IOT on resected pancreatic cancer is declining, more strict actions should be applied to achieve better outcomes. IOT is thought to be a controllable factor that can be managed, and it can be achieved not only by improving surgical technique such as minimal invasive surgery, but also by implementing an intraoperative transfusion guideline in cooperation with the anesthesiologic department.

## Figures and Tables

**Table 1:** General characteristics of the patients.

Variables	Value
Age (years)	62.5 ± 9.5
Sex	
Female	113 (39.4)
Male	174 (60.6)
Past history	
No	75 (26.1)
Yes	212 (73.9)
Symptoms	
No	57 (19.9)
Yes	230 (80.1)
Complications (Clavien-Dindo Classification)	
None	135 (47.0)
Grade 1	98 (34.1)
Grade 2	17 (5.9)
Grade 3	24 (8.4)
Grade 4	10 (3.5)
Grade 5	3 (1.0)
T status	
T0, T1, T2	262 (91.3)
T3, T4	25 (8.7)
N status	
N0	137 (47.7)
N1, N2	150 (52.3)
Preoperative CA19_9	719.6 ± 202.7
Neo CRT (Neoadjuvant chemoradiation therapy)	
No	206 (71.8)
Yes	81 (28.2)
Adj. CTx (Adjuvant chemotherapy)	
No	76 (26.5)
Yes	211 (73.5)
Tumor location	
Head	111 (38.7)
Uncinate	66 (23.0)
Neck	6 (2.1)
Body	63 (22.0)
Tail	35 (12.2)
Body + tail	6 (2.1)
LVI (Lymphovascular invasion)	
No	198 (69.0)
Yes	89 (31.0)
PNI (Perineural invasion)	
No	106 (36.9)
Yes	181 (63.1)
Resectability	
Resectable	215 (74.9)
Borderline	72 (25.1)
Surgery type	
PD	17 (5.9)
PPPD	162 (56.4)
DP	101 (35.2)
TP	7 (2.4)
Operation time (min)	392.6 ± 148.5
Combined resection	
No	194 (67.6)
Yes	93 (32.4)
Curative resection	
R0	249 (86.8)
R1	34 (11.8)
R2	4 (1.4)
EBL (mL)	654.8 ± 215.5
IOT	
No	218 (76.0)
Yes	69 (24.0)

Notes: All data are expressed as mean ± SD or N (%) Abbreviations: PD, pancreatoduodenectomy; PPPD. Pylorus preserving pancreatoduodenectomy; DP, distal pancreatectomy; TP, Total pancreatectomy; Neo CRT, Neoadjuvant chemoradiation therapy; Adj. CTx, Adjuvant chemotherapy; LVI, Lymphovascular invasion; PNI, Perineural invasion; IOT, intraoperative transfusion; and EBL, Estimated blood loss.

**Table 2:** Prognostic factors in resected pancreatic cancer: Univariate analysis.

Variables	Disease-Specific Survival (Event = 162)		Recurrence (Event = 191)	
	HR (95% CI)	*P*-Value	HR (95% CI)	*P*-Value
Age		0.476		0.505
<63	Ref		Ref	
≥63	1.12 (0.81–1.53)		0.90 (0.68–1.20)	
Sex		0.156		0.683
Male	Ref		Ref	
Female	1.36 (0.99–1.88)		1.06 (0.79–1.41)	
Past history		0.716		0.741
No	Ref		Ref	
Yes	1.06 (0.75–1.49)		0.94 (0.69–1.30)	
Symptoms		0.127		0.118
No	Ref		Ref	
Yes	1.27 (0.93–1.74)		1.24 (0.94–1.67)	
Complications		0.28		0.734
None	Ref		Ref	
Grade 1	0.88 (0.63–1.25)		0.94 (0.68–1.29)	
Grade 2	0.65 (0.30–1.41)		0.64 (0.32–1.27)	
Grade 3	0.45 (0.22–0.89)		0.64 (0.37–1.12)	
Grade 4	0.66 (0.27–1.64)		1.32 (0.64–2.73)	
Grade 5	1.04 (0.25–4.29)		0.49 (0.06–3.53)	
T status		0.033		0.482
T0/T1/T2	Ref		Ref	
T3/T4	1.75 (1.04–2.95)		1.20 (0.71–2.00)	
N status		0.002		0.668
N0	Ref		Ref	
N1/N2	1.65 (1.20–2.26)		1.05 (0.79–1.40)	
Preoperative CA19_9		0.796		0.553
<750	Ref		Ref	
≥750	1.05 (0.72–1.53)		1.10 (0.78–1.56)	
NeoCRT		0.711		0.612
No	Ref		Ref	
Yes	0.96 (0.68–1.36)		1.08 (0.79–1.48)	
Adj CTx		0.061		0.082
No	Ref		Ref	
Yes	0.68 (0.49–0.96)		0.86 (0.62–1.19)	
Tumor location		0.375		0.824
Head	Ref		Ref	
Uncinate	1.09 (0.71–1.68)		1.04 (0.71–1.51)	
Neck	2.66 (1.06–6.68)		2.80 (1.12–7.01)	
Body	0.88 (0.58–1.34)		0.71 (0.47–1.05)	
Tail	1.61 (1.01–2.57)		1.53 (0.99–2.37)	
Body + Tail	0.59 (0.18–1.88)		0.45 (0.14–1.44)	
LVI		0.084		0.292
No	Ref		Ref	
Yes	1.33 (0.96–1.84)		1.17 (0.86–1.59)	
PNI		0.597		0.393
No	Ref		Ref	
Yes	1.08 (0.79–1.49)		1.13 (0.84–1.52)	
Resectability		0.459		0.23
Resectable	Ref		Ref	
Borderline	0.86 (0.59–1.25)		1.21 (0.88–1.67)	
Surgery type		0.08		0.202
PD	Ref		Ref	
PPPD	0.58 (0.31–1.06)		1.55 (0.78–3.06)	
DP	0.56 (0.30–1.04)		1.23 (0.61–2.50)	
TP	1.23 (0.34–4.39)		4.66 (1.72–12.58)	
Operation time		0.778		0.845
<400	Ref		Ref	
≥400	1.05 (0.73–1.51)		1.03 (0.73–1.45)	
Combined resection		0.285		0.07
No	Ref		Ref	
Yes	1.19 (0.86–1.66)		1.31 (0.97–1.77)	
Curative resection		0.703		0.765
R0	Ref		Ref	
R1/R2	1.09 (0.68–1.75)		0.93 (0.59–1.45)	
EBL		0.778		0.646
<650	Ref		Ref	
≥650	1.03 (0.73–1.51)		1.06 (0.80–1.42)	
IOT		0.011		0.031
No	Ref		Ref	
Yes	1.55 (1.10–2.17)		1.41 (1.03–1.94)	
Period		0.631		0.772
2004–2007	Ref		Ref	
2008–2011	1.04 (0.71–1.52)		1.05 (0.72–1.54)	
2012–2014	0.89 (0.57–1.40)		1.67 (0.72–1.59)	

Abbreviations: PD, pancreatoduodenectomy; PPPD. Pylorus preserving pancreatoduodenectomy; DP, distal pancreatectomy; TP, Total pancreatectomy; Neo CRT, Neoadjuvant chemoradiation therapy; Adj. CTx, Adjuvant chemotherapy; LVI, Lymphovascular invasion; PNI, Perineural invasion; IOT, intraoperative transfusion; and EBL, Estimated blood loss.

**Table 3:** Prognostic factors in resected pancreatic cancer: Multivariate analysis.

Variables	Disease-Specific Survival (Event = 162)		Recurrence (Event = 191)	
	HR (95% CI)	*P*-Value	HR (95% CI)	*P*-Value
T status		0.018		
T0/T1/T2	Ref			
T3/T4	2.04 (1.13–3.68)			
N status		0.045		
N0	Ref			
N1, N2	1.46 (1.00–2.12)			
LVI		0.225		
No	Ref			
Yes	1.25 (0.86–1.82)			
Surgery type		0.126		
PD	Ref			
PPPD	0.43 (0.20–0.89)			
DP	0.41 (0.13–1.28)			
TP	0.97 (0.22–4.25)			
Adj.CTx		0.001		0.035
No	Ref		Ref	
Yes	0.51 (0.35–0.75)		0.67 (0.46–0.97)	
IOT		0.004		0.056
No	Ref		Ref	
Yes	1.94 (1.23–3.07)		1.47 (0.99–2.20)	
Combined resection				0.727
No			Ref	
Yes			0.93 (0.64–1.35)	

Abbreviations: Adj. CTx, Adjuvant chemotherapy; LVI, Lymphovascular invasion; and IOT, intraoperative transfusion.

**Table 4:** Predicting IOT in resected pancreatic cancer.

Variables	IOT		*P*-Value
	No (*n* = 218)	Yes (*n* = 69)	
Age			0.356
<63	96 (44.0)	26 (37.7)	
≥63	122 (56.0)	43 (62.3)	
Sex			0.747
Male	87 (39.9)	26 (37.7)	
Female	131 (60.1)	43 (62.3)	
Past history			0.751
No	58 (26.6)	17 (24.6)	
Yes	160 (73.4)	52 (75.4)	
Symptoms			0.189
No	53 (24.3)	4 (5.7)	
Yes	165 (75.6)	65 (94.3)	
Complications			0.293
None	101 (46.3)	34 (49.3)	
Grade 1	76 (34.9)	22 (31.9)	
Grade 2	11 (5.0)	6 (8.7)	
Grade 3	22 (10.1)	2 (2.9)	
Grade 4	6 (2.8)	4 (5.8)	
Grade 5	2 (0.9)	1 (1.4)	
T status			0.638
T0/T1/T2	200 (91.7)	62 (89.9)	
T3/T4	18 (8.3)	7 (10.1)	
N status			0.285
N0	108 (49.5)	29 (42.0)	
N1/N2	110 (50.5)	40 (58.0)	
Preoperative CA19_9			0.058
<750	178 (81.7)	49 (71.0)	
≥750	40 (18.3)	20 (29.0)	
NeoCRT			0.167
No	168 (77.1)	38 (55.1)	
Yes	50 (22.9)	31 (44.9)	
Adj CTx			0.932
No	58 (26.6)	18 (26.1)	
Yes	160 (73.4)	51 (73.9)	
Tumor location			0.065
Head	74 (33.9)	37 (53.6)	
Uncinate	50 (22.9)	20 (23.2)	
Neck	6 (2.8)	0 (0.0)	
Body	50 (22.9)	13 (18.8)	
Tail	32 (14.7)	3 (4.3)	
Body + Tail	6 (2.8)	0 (0.00)	
LVI			0.276
No	146 (67.0)	52 (75.4)	
Yes	72 (33.0)	17 (24.6)	
PNI			0.674
No	78 (35.8)	28 (40.6)	
Yes	140 (64.2)	41 (59.4)	
Resectability			0.651
Resectable	175 (80.3)	40 (58.0)	
Borderline	43 (19.7)	29 (42.0)	
Surgery type			
PD	13 (6.0)	4 (5.8)	
PPPD	114 (52.3)	48 (69.6)	
DP	87 (39.9)	14 (20.3)	
TP	4 (1.8)	3 (4.3)	
Operation time			0.064
<400	123 (56.4)	14 (20.3)	
≥400	95 (43.6)	55 (79.7)	
Combined resection			<.0.001
No	167 (76.6)	27 (39.1)	
Yes	51 (23.4)	42 (60.9)	
Curative resection			0.641
R0	188 (86.2)	61 (88.4)	
R1/R2	30 (13.8)	8 (11.6)	
EBL			<0.001
<650	145 (66.5)	10 (14.5)	
≥650	73 (33.5)	59 (85.5)	
Period			0.004
2004–2007	40 (18.3)	16 (23.2)	
2008–2011	79 (36.2)	37 (53.6)	
2012–2014	99 (45.4)	16 (23.2)	

Abbreviations: PD, pancreatoduodenectomy; PPPD. Pylorus preserving pancreatoduodenectomy; DP, distal pancreatectomy; TP, Total pancreatectomy; Neo CRT, Neoadjuvant chemoradiation therapy; Adj. CTx, Adjuvant chemotherapy; LVI, Lymphovascular invasion; PNI, Perineural invasion; IOT, intraoperative transfusion; and EBL, Estimated blood loss.
